# Implementation of a Large-Scale Ebola Vaccination Campaign in Rwanda

**DOI:** 10.3390/vaccines14070588

**Published:** 2026-07-01

**Authors:** Rosine Ingabire, Julien Nyombayire, Felix Sayinzoga, Jean Baptiste Mazarati, Amelia Mazzei, Karel Van Roey, Moses Kasigazi, Placide Nshizirungu, Oreste Tuganeyezu, Sabin Nsanzimana, Chantal Sifa, Japhet Niyonzima, Edouard Mirimo, Paula Mc Kenna, Rachel Parker, Amanda Tichacek, Jozef Noben, Kristin M. Wall, Susan Allen, Etienne Karita

**Affiliations:** 1 Center for Family Health Research, Kigali P.O. Box 780, Rwandaekarita@rzhrg-mail.org (E.K.); 2 Rwanda Biomedical Center, Kigali P.O. Box 7162, Rwanda; 3 Janssen R&D, 2340 Beerse, Belgium; 4 Gihundwe District Hospital, Rusizi P.O. Box 87, Rwanda; 5 Gisenyi District Hospital, Rubavu P.O. Box 175, Rwanda; 6 Rwanda Ministry of Health, Kigali P.O. Box 84, Rwanda; 7 Rwanda Zambia Health Research Group, Department of Pathology and Laboratory Medicine, School of Medicine, Emory University, Atlanta, GA 30322, USAkmwall@emory.edu (K.M.W.);

**Keywords:** Ebola, Ebola virus, large-scale vaccination, mass vaccination, mass immunization, Ebola vaccine, Ebola virus vaccine, Ebola hemorrhagic fever, Rwanda

## Abstract

Background/Objectives: Ebola Virus Disease (EVD) remains a public health threat in sub-Saharan Africa. The 10th Ebola outbreak in the Democratic Republic of the Congo (DRC) in 2018–2020 led the Rwanda Ministry of Health to launch a large-scale Ebola vaccination campaign using the two-dose Ad26.ZEBOV and MVA-BN-Filo regimen. The campaign was implemented by local organizations, the Center for Family Health Research and Rinda Ubuzima, in partnership with the Rwanda Biomedical Center. Methods: The campaign targeted those who live near or routinely cross the Rwanda/DRC border and unvaccinated first responders. Children <2 years and pregnant women were excluded. Results: Between December 2019 and September 2021, 219,775 individuals attended vaccination sites and 216,108 received the first dose. Of those, 110,699 (51.2%) were adults (≥18 years) and 105,409 (48.8%) were children aged 2–17 years. A total of 118,048 (54.6%) were women and 98,060 (45.4%) were men. Of all first-dose clients, 203,303 (94.1%) received the second dose. Participants who were older, male, in Rubavu district, and urban were more likely (*p* < 0.05) to be lost between the first and second dose. Most individuals who were ineligible for the second dose were women who fell pregnant after the first dose. Conclusions: Findings highlight that a large-scale vaccination campaign, including remote areas, is feasible with high adherence despite the concurrent COVID-19 pandemic. Early stakeholder engagement and local leadership were critical to success. Future studies of reasons for non-adherence, as well as strategies to integrate family planning into campaign activities to reduce ineligibility due to pregnancy, are warranted.

## 1. Introduction

Ebola Virus Disease (EVD) continues to be a public health threat across East, Central, and West Africa. Since the discovery of the first cases of EVD in 1976 in the Democratic Republic of Congo (DRC) and Sudan, over 30 outbreaks have been reported. The majority of these outbreaks have occurred in DRC, and currently, the Central and Western African countries are endemic regions [1,2]. The 2014–2016 EVD outbreak in West Africa was the world’s largest, with over 28,000 cases and more than 11,000 deaths reported. The second largest outbreak hit the North Kivu and Ituri provinces of DRC in 2018–2020, and resulted in over 3400 cases and more than 2200 deaths [3,4,5]. Following the confirmation of the first cases of Ebola in Goma, DRC, in July 2019, the World Health Organization (WHO) declared the outbreak a Public Health Emergency of International Concern (PHEIC) [5,6]. The International Health Regulations (2005) Emergency Committee for EVD in DRC recommended that at-risk countries “put in place approvals for investigational medicines and vaccines as an immediate priority for preparedness” [6,7,8]. EVD spread to Goma, a city with 2 million people at the border with Rwanda, was a particular threat to Rwanda, as more than 90,000 people were crossing the Rwanda–DRC border daily [9,10]. Therefore, Rwandan authorities decided to roll out a large-scale Ebola vaccination campaign as an urgent effort to protect its population using the Ad26.ZEBOV, MVA-BN-Filo vaccine regimen with over 200,000 doses donated by Janssen Vaccines & Prevention B.V. [4,9,11].

Large-scale vaccination feasibility and implementation in the context of an Ebola response are still unknown [12] and yet needed to guarantee the success of vaccination campaigns [13]. For example, ring vaccination deployment in response to the 2018 EVD outbreak has already confirmed that Ebola vaccination strategies may easily become ineffective if not timely and adequately executed. Missing a fifth of the targeted population or a delayed vaccine administration of just 2 weeks may make the strategy ineffective [14,15,16].

Rwanda FDA reviewed and approved use of a two-dose Ebola vaccine regimen (Ad26.ZEBOV and MVA-BN filo) under emergency use targeting 200,000 at-risk Rwandans. The campaign was named “Unprecedented Movement to drive a Unified Rwandan Initiative for National ZEBOV Immunization” (UMURINZI, meaning “Guardian” in the Rwandan national language) [11]. This paper describes the successful implementation of a large-scale Ebola vaccination campaign using a heterologous two-dose Ebola vaccine regimen.

## 2. Materials and Methods

### 2.1. Study Design, Target Population and Recruitment

This is a retrospective cohort study of adults and children of 2 years and older who participated in a large-scale Ebola vaccination campaign in Rwanda. The target population of the UMURINZI vaccination campaign was non-pregnant people ≥2 years who frequently crossed the Rwanda–DRC border, who were front-line Ebola outbreak responders not previously vaccinated against EVD, or who resided in high-risk border-proximate areas in two districts of the Western Province of Rwanda: Rubavu (bordering the city of Goma, DRC) and Rusizi (bordering the city of Bukavu, DRC). At least two months prior to the start of the program, a provincial meeting with key stakeholders was led by the Rwandan Minister of Health. Stakeholders included the Governor of the province, mayors of the districts, the head of immigration, representatives of security organizations (army and police), religious leaders and representatives of civil society organizations, and directors of district hospitals and health centers in the two districts. This meeting was followed by subsequent meetings at the district level chaired by District Mayors to locally start community engagement and launch the campaign. At least every week, the vaccination team received campaign feedback from the community via community health workers (CHWs)/recruitment teams. The district hospital director, the mayor/local leaders, and campaign representatives gave regular talks at local community meetings and on the local radio throughout the campaign to reiterate the importance of vaccination and address any questions/misconceptions from the community.

Candidates for vaccination were recruited through a range of strategies, including one-on-one invitation at border posts, door-to-door home-based invitation by CHWs, recruitment by local leaders through community gatherings, and snowball recruitment through individuals who had received the vaccine regimen. Children less than 2 years old and pregnant women were not eligible or invited for vaccination.

### 2.2. Dedicated Staff Training and Deployment

CFHR recruited and trained two physicians, one pharmacist, and one IT staff member per district as well as a total of 157 nurses before implementing the vaccination activities. The trainings were provided by CFHR clinicians, a pharmacist, and a data/IT specialist and included a 2-day didactic session followed by a 3-day practical training. The training topics focused on: (i) how to deliver Ad26.ZEBOV, MVA-BN-Filo Ebola vaccination information to clients using the Rwanda FDA-approved factsheet; (ii) use of vaccination logbooks, the biometric-based vaccination monitoring system, and vaccination databases; (iii) vaccine handling, storage, and administration; and (iv) safety monitoring and anaphylaxis/allergy emergency management planning.

### 2.3. Vaccination Site Settings

**Figure 1:**
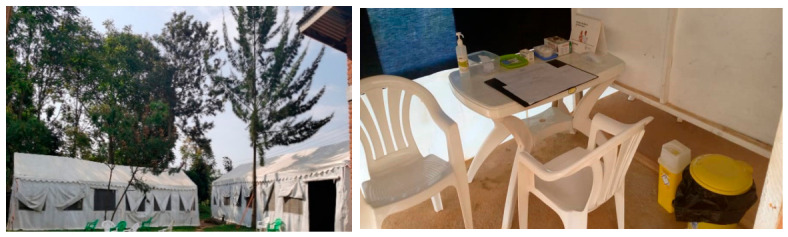
UMURINZI vaccination site with two tents showing different rooms and a vaccination room setting.

Twelve health centers, six in Rubavu and six in Rusizi, two border posts in Rubavu controlling movement between Rwanda and DRC, and the Kigali International Airport were selected to serve as vaccination sites. Tents with partitions were installed at vaccination sites to provide extra space, and a local server using a 4G internet connection was installed to allow timely recording and reporting of vaccination activities to a central server and synchronization with other sites’ data. Extra refrigerators/freezers were purchased and calibrated as needed. The vaccination sites consisted of a waiting area, a reception area, at least five rooms for vaccine administration, a pharmacy, and a room for post-vaccine observation (Figure 1).

### 2.4. Vaccine Regimen

The Ebola vaccine regimen consisted of 0.5 mL at a dose of 5 × 10^10^ viral particles of Ad26.ZEBOV vaccine given via intramuscular (IM) administration of an injection into the deltoid muscle in the upper arm at the first visit followed by 0.5 mL of MVA-BN-Filo vaccine at a dose of 1 × 10^8^ infectious units through IM injection approximately eight weeks later, with a recommended window period of 42 days post dose 1 up to 85 days.

Ad26.ZEBOV (Zabdeno) is a non-replicating recombinant human adenovirus serotype 26 vector encoding the Ebola virus Mayinga glycoprotein. MVA-BN-Filo (Mvabea) is a recombinant Modified Vaccinia Ankara (MVA) vector encoding glycoproteins from Ebola, Marburg, Sudan, and Taï Forest viruses, as well as Ebola nucleoprotein. As non-replicating viral vector vaccines, both were contraindicated in pregnancy at the time of campaign launch due to precautionary exclusions in the absence of established safety data in pregnant women. This biological basis underlies the eligibility requirements described in Section 2.5 and Section 2.6, including mandatory pregnancy testing and exclusion of pregnant participants.

### 2.5. Vaccine Supply and Cold Chain

Janssen trained the Rwanda Biomedical Center (RBC) and CFHR team on Ebola vaccine supply and cold chain procedures prior to any shipment in Rwanda. Ebola vaccines were shipped monthly by Janssen from their stockpile storage sites in Belgium to the RBC for storage in a temperature-monitored cold room in Kigali. From there, vaccines were then transported to Rubavu and Rusizi district hospital pharmacies by car (4–6 h driving distance, respectively) for weekly distribution to vaccinating health centers/sites. The vaccines were transported in a frozen state (−20 °C) using shipping boxes with pre-conditioned cold packs with data loggers to monitor the temperature every 10 min, and at least five calibrated freezers (−20 °C) were used for storage at each district pharmacy. At the health center pharmacy, the vaccines were kept at 2 °C to 8 °C in WHO-pre-qualified ice-lined refrigerators with one generator installed for electricity backup. Daily temperature checks were done by local and district pharmacists with timely support from the quality assurance team composed of CFHR and Janssen staff.

### 2.6. Education Sessions and Vaccine Administration

At the first visit, a group education session was conducted in the waiting area using a Rwanda FDA-approved factsheet. This factsheet provided information on Ebola infection, type of Ebola vaccines to be used and when/how to receive them, eligibility, and expected side effects for children and adults. Flipcharts with clear and self-explanatory images were used to disseminate key information from the factsheet to participants. During this session, family planning (FP) counseling on all modern methods was introduced to support clients/couples to avoid pregnancy between vaccine doses. This allowed interested clients to think about their choice while in the waiting area, discuss further, and make informed decisions about FP methods.

After the group education session, individuals were received one by one in individual rooms and they were given an opportunity to ask additional questions before proceeding with eligibility screening. Eligibility criteria included: living/working in the vicinity of an Ebola outbreak, such as people living in Rusizi and Rubavu districts; being at least 2 years old; being available for at least 2 months post first dose for the second-dose visit; a negative pregnancy test for childbearing participants; and absence of known allergy to Ebola vaccine regimen ingredients. Eligible participants who provided verbal consent were registered by providing a biometric iris scan and a digital photograph for future identification (ID) [17], and then they were vaccinated and given an UMURINZI vaccination card.

At the second vaccination visit, UMURINZI card and biometric data were checked and people without any severe allergic reaction and a negative pregnancy test (for those of childbearing potential) were eligible to receive the second dose.

Each vaccinated individual was observed for 15 min before being released, and FP methods were provided to clients during this period or before client exit. For logistical reasons, all FP methods were mainly given at the health center hosting the vaccination activities; while two vaccination sites at border crossing that did not have co-located health centers offered only oral contraceptives. We allowed those attending more than 85 days after dose one to still receive the second vaccine.

### 2.7. Data Collection

Each participant was assigned a unique ID number linked with their vaccination recipient data including age, year of birth, gender, eligibility assessment, vaccination dates, and vaccine vial/batch number. The campaign used a vaccination monitoring platform (VMP) which consisted of: (i) a biometric system using an iris scan and a photograph to identify a client; (ii) mobile messaging technology using a recorded client telephone number to send reminders via automated short message service (SMS) and recorded voice messages; and (iii) an online dashboard called “Power BI (Business Intelligence)” showing enrolment, adherence and individual vaccination details for timely and adequate support of the vaccination [17].

Additional data related to inclusion/exclusion criteria and safety were recorded electronically in SurveyCTO (Dobility, CA, USA) using Android tablets. The data were exported into Excel and cleaned and analyzed using STATA 15 Software.

### 2.8. Data Analysis

We describe the number and proportion of patients received in UMURINZI, those found ineligible, and reasons for ineligibility. Among those found eligible, we describe the distribution of those who received the first dose and second dose, reasons for ineligibility for the second dose, and loss to follow-up.

We also describe the cumulative number of clients receiving the first and second dose in the UMURINZI large-scale Ebola vaccination campaign over the December 2019–September 2021 campaign time period, noting major events related to the COVID-19 pandemic and the national lockdown response.

Participants characteristics collected during the campaign (age, gender, district, and urban or rural vaccination site area) were described using counts and percentages, overall and stratified by dose received (first or second). Although the majority of the Rubavu and Rusizi districts is considered rural [18,19], vaccination sites located in areas with modern infrastructure, public services, commercial activities, and private sector services were considered urban as they were expected to serve mainly urban people; rural vaccination sites were defined as those located in the remaining/remote areas of the districts where agricultural and livestock activities are prevalent [20].

Adherence to the two-dose Ebola regimen, defined as clients who received both the first and second Ebola vaccine doses, was calculated overall and by district. Finally, the associations between participant characteristics (age, gender, district, and urban or rural vaccination site area) and non-adherence to the two-dose vaccine regimen, defined as clients who received the first dose only, including first-dose clients who did not come back for the second dose as well as those who came back for the second dose but were found to be ineligible to receive the vaccine, were estimated using a multivariable logistic regression model. The logistic regression model estimated adjusted odds ratios (AOR) and 95% confidence intervals (CIs). Site-level clustering was not accounted for and may affect the precision of confidence intervals. The model’s discriminatory performance was assessed using area under the receiver operating characteristic curve (AUC).

Kaplan–Meier methods were used to estimate time to second-dose completion, defined as the interval between first-dose administration and second-dose receipt. Individuals who did not receive a second dose were censored at the study end date. The curves were compared between the pre-COVID-19 period and during-COVID-19 period using the log-rank test.

The missing data were minimal (<0.1% of records), and records with missing values were excluded from their specific analyses in which those variables were required.

For clarity, we distinguish between return rate (the proportion of dose-1 recipients who attended a second-dose visit: 203,834/216,108 = 94.3%) and completion rate (the proportion who actually received dose 2: 203,303/216,108 = 94.1%). Individuals who returned but were found ineligible for dose 2 (*n* = 531, primarily due to pregnancy) are captured in the return rate but excluded from the completion rate. The 94% figure reported in the abstract and results refers to the completion rate.

### 2.9. Ethical Considerations

The Ad26.ZEBOV, MVA-BN-Filo Ebola vaccine regimen was approved by Rwanda Food and Drug Authority for emergency use in our campaign [11]. Rwanda National Ethics Committee determined the UMURINZI campaign to be a non-research program, thus not requiring ethical clearance, and that written consent was not required. Only verbal consent was obtained and documented in electronic records (SurveyCTO) at the time of each visit. All clients under 18 years old were required to be accompanied to the vaccination site, and verbal consent was obtained from the parent or legal guardian accompanying them. De-identified data were recorded and analyzed.

## 3. Results

### 3.1. The Launch and Scale-Up Process for the UMURINZI Large-Scale Ebola Vaccination Campaign

The campaign was launched by the Minister of Health in December 2019 and was completed in September 2021. Many influential people and stakeholders in the target population including local leaders were willing to be among the first clients to be vaccinated, and most of the time, this was done publicly during launching events.

**Figure 2:**
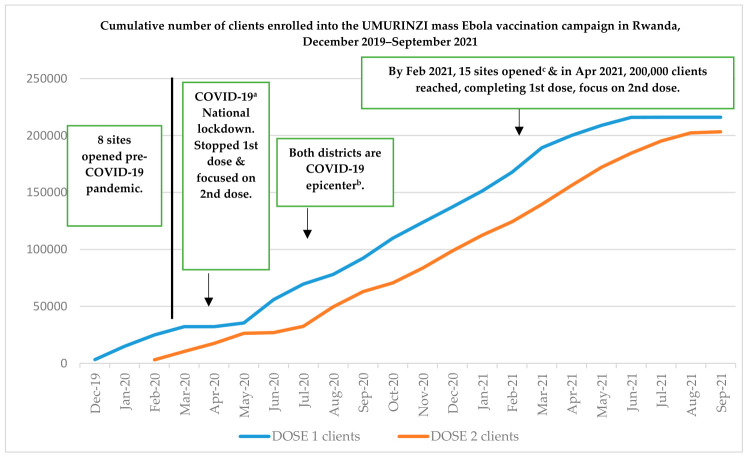
Cumulative number of clients enrolled into the UMURINZI large-scale Ebola vaccination campaign in Rwanda, December 2019–September 2021. a: COVID-19 national lockdown from 21 March 2020 to 4 May 2020. b: Three vaccination sites suspended their activities—2 sites at the border post in Rubavu district completely closed and 1 site in Rusizi temporarily turned into a COVID-19 treatment center. c: COVID-19 mitigation plan involved (i) opening new sites, (ii) redirecting clients from closed sites to nearby operational sites, and (iii) increasing vaccination site capacity in terms of space and/or staff.

Figure 2 shows the evolution of vaccination activities over time. We progressively opened eight vaccination sites to launch the campaign. Three months into the campaign, the vaccination activities were interrupted due to the COVID-19 outbreak in March 2020, which led to the first national lockdown from 21 March 2020 to 4 May 2020. By that time, more than 30,000 individuals had received the first Ebola vaccine dose, and a third of them had already received their second dose across eight sites. The lockdown stopped all first-dose activities and closed one vaccination site at the DRC border, “la Corniche site”, but later allowed the campaign to focus on the second-dose vaccinations. The second COVID-19 lockdown (July 2020–August 2020) did not significantly affect the vaccination program. This period was followed by steady Ebola vaccine provision with doubled uptake (from about 80,000 first-dose clients as of August 2020 to more than 160,000 clients by February 2021) after an accelerated COVID-19 mitigation plan that allowed the team to fully vaccinate ¾ of all clients by February 2021. The COVID-19 mitigation plan involved: (i) opening new sites, (ii) redirecting clients from closed sites to nearby operational sites, and (iii) increasing vaccination site capacity in terms of space and/or staff.

### 3.2. Characteristics of Clients Attending the UMURINZI Large-Scale Ebola Vaccination Campaign

**Figure 3:**
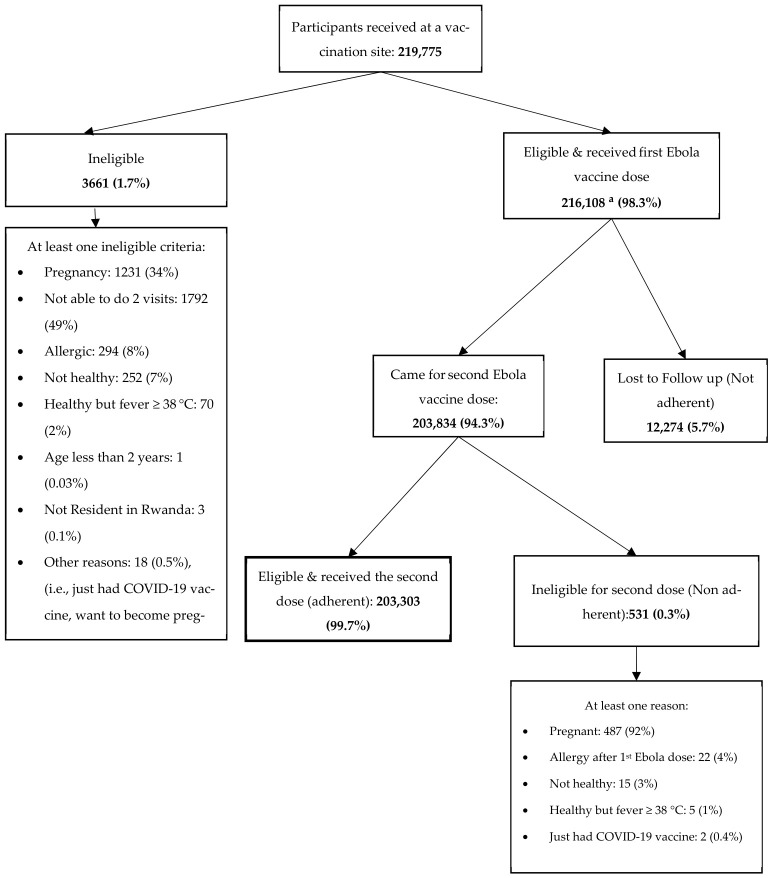
Flow diagram showing vaccination flow and eligibility assessment for the UMURINZI large-scale vaccination campaign in Rwanda, December 2019–September 2021. ^a^: During analysis, 6 records with invalid first-dose dates or second-dose dates were removed from our dataset assessing Ebola vaccine adherence in our campaign.

From December 2019 to September 2021, a total of 219,775 individuals attended Ebola vaccination sites, of whom 216,108 (98%) received their first Ad 26.ZEBOV vaccine (Figure 3, Supplementary Table S1). Of the 3661 individuals who were not vaccinated, 1792 (49%) reported that they would not be available for the second dose, and 1231 (34%) were women with a positive pregnancy test. Of those who received their first dose, 203,834 (94%) came for a second dose and 203,303 (94%) received the second dose. Most individuals who were ineligible for the second dose were women who fell pregnant after the first dose (92%).

As shown in Table 1, among 216,108 who received the first Ebola vaccine, 110,699 (51%) were adults aged 18 years or more; 42,908 (20%) were 12–17 years; 40,700 (19%) were 6–11 years; and 21,801 (10%) were 2–5 years old. A total of 118,048 (55%) of those who received the first Ebola vaccine were female. The majority of clients who received the first dose (153,893; 71.2%) were vaccinated at a rural vaccination site.

### 3.3. Adherence to the Two-Dose Ebola Vaccine Regimen

**Figure 4:**
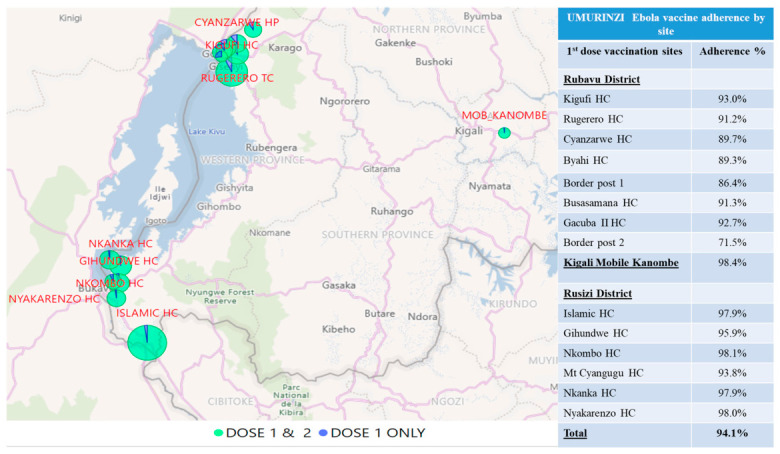
The adherence to the 2-dose regimen by vaccination site during the UMURINZI large-scale Ebola vaccination campaign in Rwanda, December 2019–September 2021. HC: health center. Adherence is defined as clients who received both the first and second Ebola vaccine doses (*N* = 203,303).

Adherence to the two-dose Ebola vaccination regimen, defined as clients who received both the first and second Ebola vaccine doses, was 94% (203,303/216,108) (Figure 3 and Figure 4). Nearly half of all vaccination sites had more than 94% adherence, and most of the sites (12/15 sites) had adherence of more than 90%. One vaccination site, “Byahi health center”, had 89.3%, and two sites (Border post 1 and Border post 2), both located at the border post in Rubavu district, had the lowest second-Ebola-vaccine adherence values of 86.4% and 71.5%, respectively.

In general, the majority of fully vaccinated clients received their second dose within their window period (89%; 180,751) and the people receiving both doses at the same site were more likely to be adherent to the second-vaccine schedule than those who received both doses at different sites (90% vs. 68%, *p* < 0.01).

### 3.4. Time to Second Dose and Factors Associated with Non-Adherence to a Two-Dose Ebola Vaccine Regimen

Kaplan–Meier curves (Supplementary Figure S1) showed a significantly higher cumulative probability of second-dose completion among individuals initiating vaccination during the COVID-19 period compared with those initiating vaccination before the COVID-19 period (the log-rank test, *p* < 0.01). However, after adjustment, the COVID-19 period was not associated with second-dose non-adherence, as shown in Table 2. Table 2 also shows that older age (≥18 vs. 2–5 years and 12–17 vs. 2–5 years), being male vs. female, being in Rubavu vs. Rusizi, and being in an urban area vs. a rural area were independently and statistically significantly (*p* < 0.05) associated with non-adherence in our large-scale vaccination campaign.

## 4. Discussion

Following 2019 WHO declaration of the 10th DRC Ebola outbreak as a PHEIC, a pre-emptive large-scale Ebola vaccination campaign was initiated in Rwanda and reached its target of 200,000 people successfully completing the two-dose Ebola vaccine regimen. Among people receiving dose 1, 94% returned and received their second dose, indicating very good adherence to the vaccine regimen. Our implementation strategies suggest that early involvement of Rwandan National Stakeholders up to local leaders resulted in massive population engagement and vaccine uptake despite the COVID-19 pandemic. Furthermore, appointment reminder SMS or voice recording messages in Kinyarwanda, as well as data-driven tracking and follow-up, may also have helped maintain higher adherence levels. This campaign was also facilitated by dedicated staff and efficient logistics, as well as an innovative platform to track enrolment and adherence. Despite FP service opportunities at the vaccination sites, pregnancy was an ineligibility criterion for vaccination in this campaign, and it was the main reason why people returning for their second dose were not vaccinated [21].

Our study confirmed the feasibility of rolling out a two-dose Ebola prophylactic vaccination campaign using a conditionally approved Ebola vaccine regimen in an LMIC, exceeding the targeted at-risk population. Similar to our study, other studies have found that an early and coordinated multi-level stakeholder engagement successfully promoted the participation of the community in a vaccination campaign as well as supporting the implementing health team [22,23,24].

In our study, strong stakeholder support and good coordination and adaptation of the implementation strategies were able to minimize the impact of the COVID-19 outbreak on the UMURINZI campaign, thus highlighting the capacity of rolling out more than one outbreak response with minimum interference, as was recommended later by WHO [24,25,26]. Other countries suffered from long-term vaccination interruptions due to COVID-19, which exposed them to the risk of preventable infectious diseases post COVID-19 [27,28]. It was noted that in more than 50 countries, about 60% of vaccination campaigns were disrupted at the beginning of COVID-19, which resulted in about 800 million people postponing or missing their vaccinations [28]. The majority were African countries for reasons such as unavailability of public health guidance on safe large-scale vaccination rollout; interruption of product supply due to travel restrictions or fear for new infection when going for vaccination; and decreased access to sites with travel restrictions [28,29].

While multi-dose vaccine regimens are known to have lower adherence, compared to single-dose vaccination [30,31,32], our findings have shown that a higher adherence rate to a two-dose vaccine regimen can be achieved in a large-scale vaccination in an LMIC. It is possible that our novel biometric-based vaccination monitoring platform used to track and remind clients of their second-dose schedule may have contributed to enhancing adherence in our Ebola vaccination campaign [17,33,34,35].

Our study utilized implementation methods that could be used for future large-scale Ebola vaccination campaigns with a two-dose vaccine regimen to enhance the adherence to the second dose in LMICs.

Contrary to a recent study [36], which highlights that women were less adherent to a full-dose vaccination than their male counterparts in LMICs, our results demonstrated that male participants were less likely to be adherent compared to female participants. Per a recent study, one reason could be that men consider themselves at low risk of getting the disease [24]. This, however, would be particularly concerning as the male population could be more exposed to emerging infectious diseases while working outside their homes, and most importantly, for Ebola, the virus has been shown to persist in male survivors [37,38,39,40].

The absence of a significant difference in non-adherence between the 2–5 and 6–11 age groups (AOR 0.94, *p* = 0.20), in contrast to the significantly higher non-adherence among adolescents and adults, likely reflects caregiver dependency: children in both younger groups rely on a parent or guardian to bring them for both doses, whereas adolescents and adults have greater scheduling autonomy and competing demands. This finding underscores the importance of caregiver engagement and SMS reminder systems for multi-dose pediatric vaccination programs.

Contrary to other studies that reported that poor adherence would be associated with campaigns implemented in rural areas [41], our study found that adherence was lower in urban areas than in rural regions. Although the good adherence in rural regions is encouraging, since the Ebola vaccine would be needed more in remote areas that are considered at risk of Ebola outbreaks, it would be important to reach similar adherence in urban areas which are more populated and would be prone to spread Ebola infection to other regions countrywide or to other countries [6,42,43,44]. It is important that future studies investigate the reasons for lower adherence in urban areas and male clients and suggest tailored implementation strategies that could better address and maximize adherence in these situations. However, as other studies have found [36], given our limited data on clients’ characteristics, it is also possible that our observed geographic difference could have been influenced or explained by other confounders such as income and individual distance to vaccination centers. Although the COVID-19 pandemic did not significantly impact the overall adherence rate for this campaign, it is noteworthy that two sites at the border post (Border post 1 and Border post 2) were closed for 9 months and 14 months, respectively, and had the lowest adherence rate in our campaign despite redirecting clients to their nearby vaccination center during the lockdown/COVID-19 period. One possible reason for this is that the interruption of trading activities on the border due to the lockdown meant that people were no longer regularly crossing the border with DRC and they might have lost interest in completing Ebola vaccination [24]. Notably, the proportion of clients lost to follow-up for the second dose was slightly lower during COVID-19 restrictions compared to the pre-restriction period (5.7% vs. 8.0%, *p* < 0.01), suggesting that the pandemic mitigation strategies were effective in maintaining overall adherence. Of all second-dose recipients, 22,493 (11%) received dose 2 after the recommended 85-day window; these late doses were accepted per protocol, as described in Section 2.6.

Pregnancy was an ineligibility criterion for vaccination in this campaign; more women were excluded from dose 2 vaccination because they were found to be pregnant. Previous Ebola vaccination interventions, including clinical trials, showed that Ebola prevention is an unmet need in pregnant women [24,45]. While the rest of the population has been benefiting from Ebola vaccine protection, pregnant women continued to be left at risk despite the near certainty that, if infected, they would have an extremely high risk of maternal–child mortality [45,46,47]. In consideration of this, a phase 3 clinical trial in pregnant women was initiated in the UMURINZI campaign areas, just 10 months after launching the campaign, in order to assess the safety of this two-dose Ebola vaccine regimen in pregnancy, hence facilitating pregnant women’s access to Ebola prophylaxis [48,49]. Our findings, which show that pregnancy is the main reason for dose 2 ineligibility in people who received the first dose, also highlight that if pregnant women continue to be excluded from vaccination, this could be a limiting factor in achieving high adherence to the vaccine regimen in Africa and LMICs where contraceptive methods might not be accepted or used [50,51,52]. Therefore, any effort to provide and increase FP uptake during large-scale vaccination should be undertaken. In a recent analysis, we reported that most women who became pregnant after dose 1 never received dose 2 after pregnancy completion, and that contraceptive uptake was low prior to and during the campaign [21]. The exclusion of pregnant women from this vaccination campaign reflects a regulatory requirement at the time—not a programmatic choice—given the absence of established safety data in pregnant women. Integration of family planning services into vaccination campaigns should prioritize preventing pregnancy until vaccination is complete while ensuring informed choice, reproductive autonomy, and rights-based counseling. Importantly, women should not be excluded from multi-dose vaccine or drug campaigns solely based on non-uptake of family planning when pregnancy is a contraindication.

Limitations: Several limitations of this study warrant acknowledgment. First, a key limitation is that the UMURINZI campaign was implemented as a public health program rather than a research study, resulting in only limited individual-level demographic and behavioral data collection. Important potential determinants of vaccine adherence—including education level, socioeconomic status, occupation, distance to vaccination site, and frequency of border crossing—were not routinely captured. Consequently, residual confounding of the observed associations cannot be excluded; all reported associations should therefore be interpreted as descriptive rather than causal. Future studies should investigate other predictors of vaccine adherence to more fully understand the factors influencing two-dose vaccination completion and to strategically optimize uptake and adherence in future large-scale Ebola vaccination campaigns in Africa and other low- and middle-income settings. Second, pregnancy exclusion reflects the regulatory status of both Ad26.ZEBOV and MVA-BN-Filo at the time of campaign launch, as safety data in pregnant women were not yet available; this is not a programmatic choice but a vaccine regulatory requirement. Third, the UMURINZI campaign targeted a specific high-risk population—individuals living near or routinely crossing the Rwanda–DRC border and unvaccinated first responders—and used recruitment strategies including snowball sampling that may have preferentially enrolled more motivated individuals. The adherence rates observed here may not be generalizable beyond high-risk border communities with comparable levels of stakeholder engagement and resource investment. Fourth, the Kigali International Airport site enrolled a small, highly selected population (n = 870, <0.5% of total enrollees), and results for this site should be interpreted with caution. Fifth, the regression model did not account for clustering of individuals within vaccination sites, which may affect the precision of confidence intervals. Sixth, while the biometric monitoring platform was a key innovation, limitations include potential iris scan failures, connectivity issues at remote sites, and dependency on clients having a registered telephone number for SMS reminders.

## 5. Conclusions

Involvement of national stakeholders and local leaders before and during a large-scale Ebola vaccination was key for community uptake of the Ebola vaccine and to guide the Ebola vaccination team through the COVID-19 pandemic period. Two-dose vaccine regimens can be rolled out successfully in Africa or LMICs and achieve higher adherence if adequate and dedicated resources and effective logistics are used, including novel platforms like the biometric-based vaccination monitoring platform to improve timely tracking and easy access to vaccination sites in remote regions. As researchers work hard to develop vaccines to control an infectious disease outbreak, similar efforts and urgency should be used to obtain safety data in pregnant women as they are a vulnerable population, especially if directly affected by the emerging pathogen. Our findings show that a successful large-scale vaccination in Africa, including remote areas, is possible and will help to inform vaccine roll-out strategies, especially when using a two-dose vaccine regimen or when confronted with a concurrent outbreak, like COVID-19. 

## Figures and Tables

**Table 1:** The characteristics of clients attending the UMURINZI large-scale Ebola vaccination in Rwanda, December 2019–September 2021.

	Total Received at a Vaccination Site		Received 1st Dose		Received 2nd Dose	
	*N* = 219,775	Col%	*N* = 216,108	Col%	*N* = 203,303	Col%
Age						
2–5	22,068	10.0	21,801	10.1	21,028	10.3
6–11	40,972	18.6	40,700	18.8	39,320	19.3
12–17	43,245	19.7	42,908	19.9	40,356	19.9
≥18	113,490	51.6	110,699	51.2	102,599	50.5
Gender						
Female	121,062	55.1	118,048	54.6	111,852	55.0
Male	98,713	44.9	98,060	45.4	91,451	45.0
District						
Kigali International Airport	878	0.4	870	0.4	856	0.4
Rubavu	107,004	48.7	105,420	48.8	95,616	47.0
Rusizi	111,893	50.9	109,818	50.8	106,831	52.6
Vaccination site area						
Rural	155,643	70.8	153,893	71.2	146,355	72.0
Urban	64,132	29.2	62,215	28.8	56,948	28.0

**Table 2:** Factors associated with non-adherence to the 2nd-dose Ebola vaccine in the UMURINZI mass Ebola vaccination campaign in Rwanda, December 2019–September 2021.

	Non-Adherent	Adherent	Adjusted Odds Ratio	95% CI		*p*-Value
	*N* (row %)	*N* (row %)		Lower limit	Upper limit	
	12,805	203,303				
Age						
2–5	773 (3.5)	21,028 (96.5)	1			
6–11	1380 (3.4)	39,320 (96.6)	0.94	0.86	1.03	0.196
12–17	2552 (5.9)	40,356 (94.1)	1.48	1.36	1.61	<0.01
≥18	8100 (7.3)	102,599 (92.7)	1.86	1.72	2.01	<0.01
Gender						
Female	6196 (5.3)	111,852 (94.7)	1			
Male	6609 (6.7)	91,451 (93.3)	1.32	1.27	1.37	<0.01
District						
Rusizi	2576 (2.4)	107,244 (97.6)	1			
Rubavu	9686 (9.2)	95,738 (90.8)	3.35	3.21	3.50	<0.01
Kigali international Airport	14 (1.6)	856 (98.4)	0.33	0.20	0.57	<0.01
Vaccination site area						
Rural	7538 (4.9)	146,355 (95.1)	1			
Urban	5267 (8.5)	56,948 (91.5)	1.34	1.31	1.41	<0.01
COVID-19 timing						
Pre-COVID-19 period	2586 (8.0)	29,572 (92.0)	1	-	-	
During COVID-19	10,219 (5.6)	173,731 (94.4)	1.05	1.00	1.11	0.07

CI: confidence interval. Non-adherence (*N* = 12,805) is defined as clients who received the first dose only. These include first-dose clients who did not come back for the second dose (*N* = 12,274) as well as those who came back for the second dose but were found to be ineligible to receive the vaccine (*N* = 531). Adherence is defined as clients who received both the first and second Ebola vaccine doses (*N* = 203,303).

## Data Availability

De-identified, individual participant data, a data dictionary, and author-generated code are available via Harvard Dataverse (https://dataverse.harvard.edu/, accessed on 17 October 2024) at https://doi.org/10.7910/DVN/CZ8W3J (accessed on 17 October 2024). Access will be permitted pending a signed data use agreement.

## Supplementary Materials

The following supporting information can be downloaded at https://www.mdpi.com/article/10.3390/vaccines14070588/s1. Figure S1: Kaplan-Meier curves for the two dose vaccine completion adherence before and during the COVID-19 pandemic; Table S1: UMURINZI total participants vaccinated by site.

## Abbreviations

The following abbreviations are used in this manuscript:

AUC	Area under the receiver operating characteristic curve
CFHR	Center for Family Health Research
CHWs	Community health workers
DRC	Democratic Republic of the Congo
EVD	Ebola Virus Disease
FDA	Food and Drug Administration
FP	Family planning
ID	Identification
IM	Intramuscular administration
LMIC	Low- and Middle-Income Countries
PHEIC	Public Health Emergency of International Concern
RBC	Rwanda Biomedical Center
UMURINZI	Unprecedented Movement to drive a Unified Rwandan Initiative for National ZEBOV Immunization
WHO	World Health Organization
