# Supplementary material for: Implementation of a Large-Scale Ebola Vaccination Campaign in Rwanda
Source: Vaccines (Basel). 2026 Jul 1;14(7):588. doi: 10.3390/vaccines14070588 (PMC13416593; doi:10.3390/vaccines14070588)
Supplement: Supplementary file 1 [file vaccines-14-00588-s001.zip › Supplement Table S1_UMURINZI TOTAL PARTICIPANT VACCINATED BY SITE.pdf]

Supplement Table S1: UMURINZI TOTAL PARTICIPANTS VACCINATED BY SITE

1st dose vaccination sites	Received 1st dose	Attended 2nd dose visit	Received 2nd dose	Adherence %
**Rubavu District**				
Kigufi HC	31,958	29,733	29,729	93.0%
Rugerero HC	17,405	15,901	15,869	91.2%
Cyanzarwe HC	13,118	11,789	11,765	89.7%
Byahi HC	12,526	11,183	11,180	89.3%
Poids Lourds	11,598	10,045	10,025	86.4%
Busasamana HC	9,423	8,626	8,599	91.3%
Gacuba II HC	8,196	7,602	7,594	92.7%
La Corniche	1,196	855	855	71.5%
**Kigali Mobile Kanombe**	870	858	856	98.4%
**Rusizi District**				
Islamic HC	43,204	42,418	42,277	97.9%
Gihundwe HC	15,305	14,673	14,624	95.9%
Nkombo HC	14,399	14,157	14,119	98.1%
Mt Cyangugu HC	8,434	7,953	7,914	93.8%
Nkanka HC	16,639	16,368	16,291	97.9%
Nyakarenzo HC	11,837	11,673	11,606	98.0%
**Total**	**216,108**	**203,834**	**203303**	**94.1%**

HC: Health Center
