# Supplementary figures and images for: Implementation of a Large-Scale Ebola Vaccination Campaign in Rwanda
Source: Vaccines (Basel). 2026 Jul 1;14(7):588. doi: 10.3390/vaccines14070588 (PMC13416593; doi:10.3390/vaccines14070588)

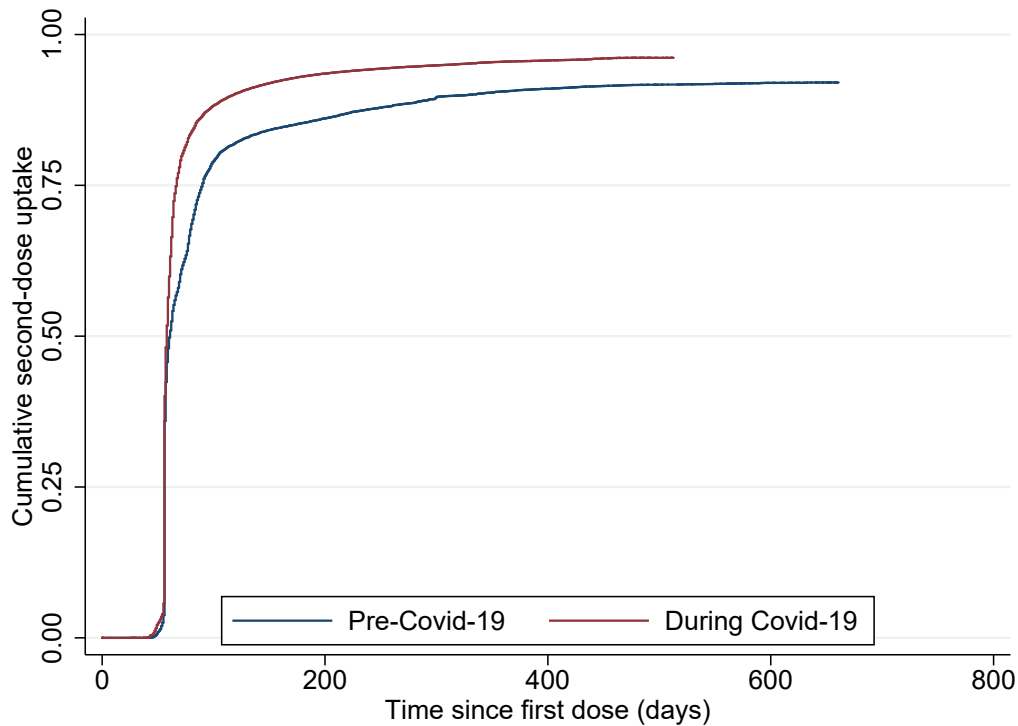

Supplement: Supplementary file 1 [file vaccines-14-00588-s001.zip › Figure S1. Kaplan Meier with Covid-19 period.pdf]
